# Supplementary figures and images for: Comprehensive Transcriptome Analysis Uncovers Distinct Expression Patterns Associated with Early Salinity Stress in Annual Ryegrass (Lolium Multiflorum L.)
Source: Int J Mol Sci. 2022 Mar 18;23(6):3279. doi: 10.3390/ijms23063279 (PMC8948850; doi:10.3390/ijms23063279)

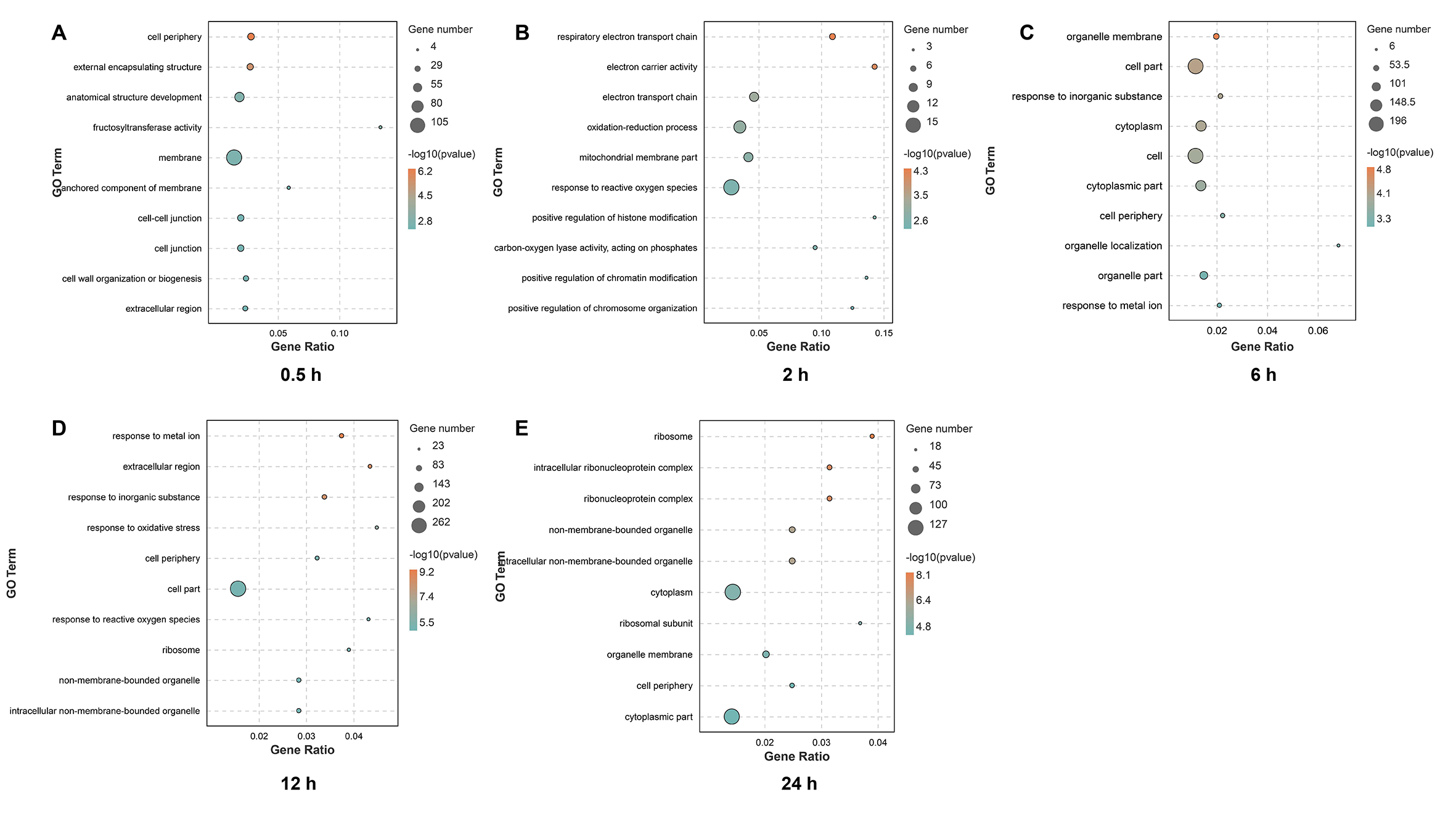

Supplement: Supplementary file 1 [file ijms-23-03279-s001.zip › Supplementary Tables and Figures/Supplementary Fig S1.tif]

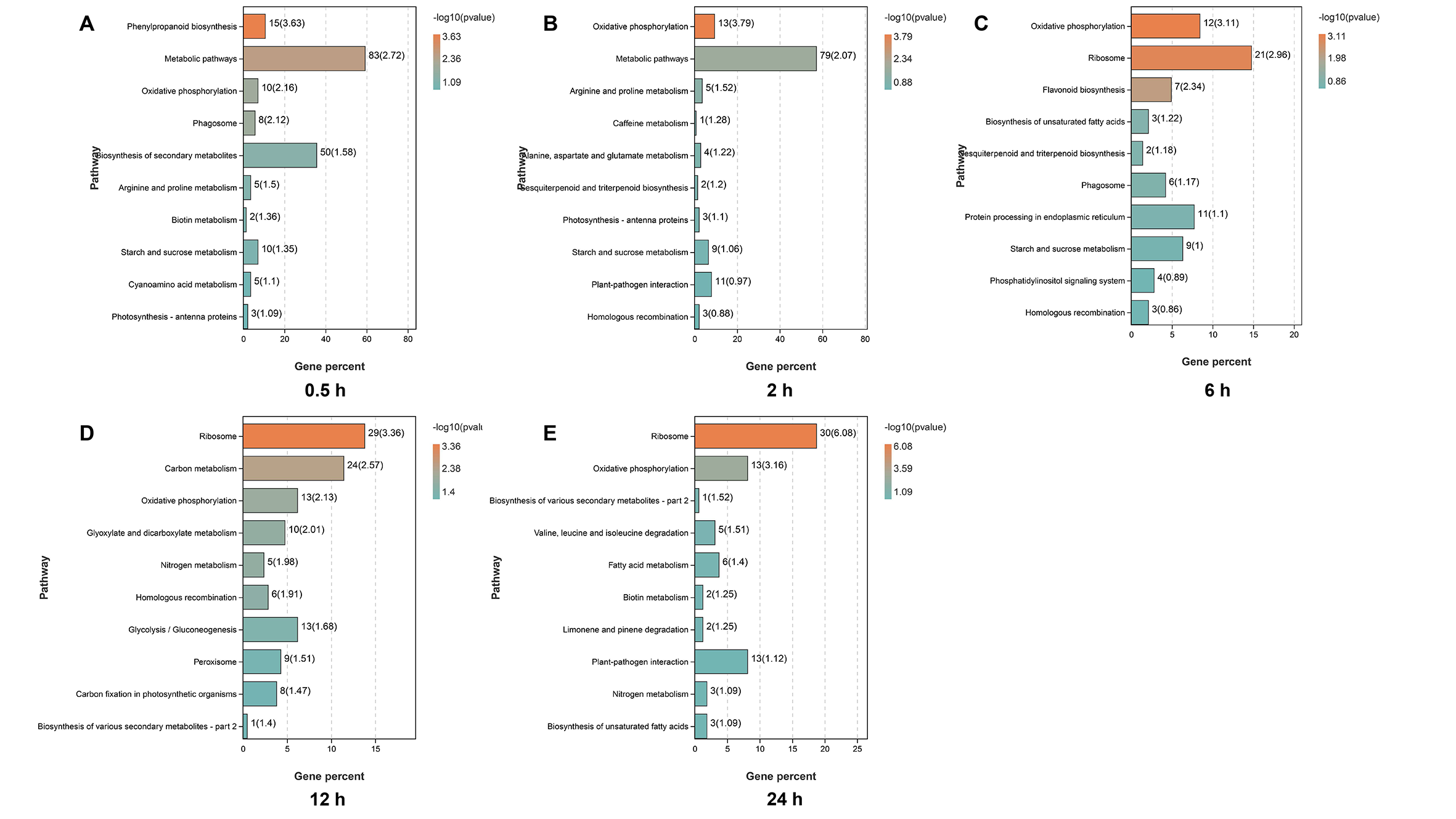

Supplement: Supplementary file 1 [file ijms-23-03279-s001.zip › Supplementary Tables and Figures/Supplementary Fig S2.tif]

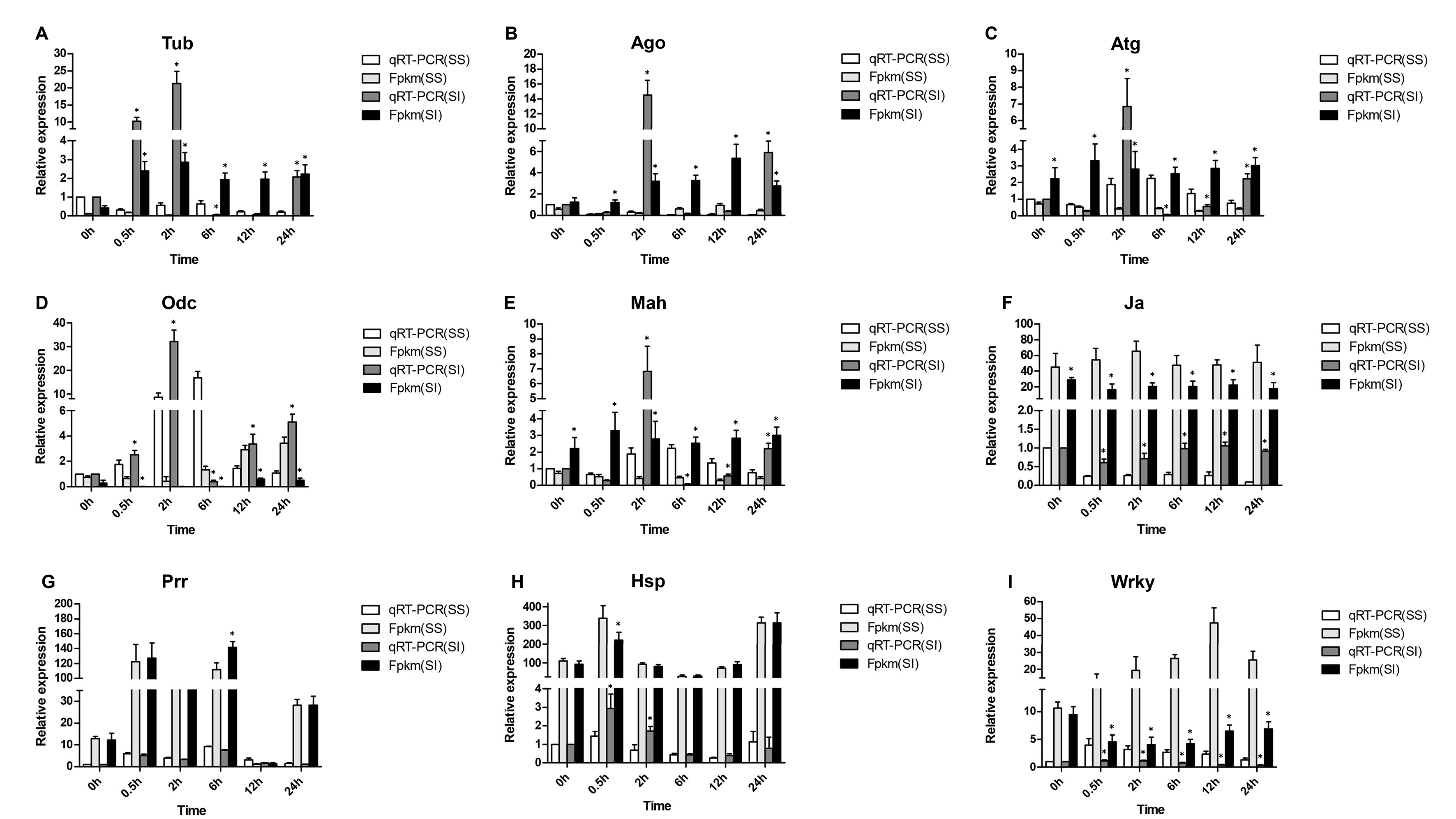

Supplement: Supplementary file 1 [file ijms-23-03279-s001.zip › Supplementary Tables and Figures/Supplementary Fig S3.tif]
